# Epstein Barr virus Latent Membrane Protein-1 enhances dendritic cell therapy lymph node migration, activation, and IL-12 secretion

**DOI:** 10.1371/journal.pone.0184915

**Published:** 2017-09-14

**Authors:** James M. Termini, Sachin Gupta, Francesca N. Raffa, Elizabeth Guirado, Margaret A. Fischl, Liguo Niu, Saravana Kanagavelu, Geoffrey W. Stone

**Affiliations:** 1 Department of Microbiology and Immunology, Miami Center for AIDS Research and the Sylvester Comprehensive Cancer Center, University of Miami Miller School of Medicine, Miami, FL, United States of America; 2 Department of Medicine and Miami Center for AIDS Research, University of Miami Miller School of Medicine, Miami, FL, United States of America; Swedish Neuroscience Institute, UNITED STATES

## Abstract

Dendritic cells (DC) are a promising cell type for cancer vaccines due to their high immunostimulatory capacity. However, improper maturation of DC prior to treatment may account for the limited efficacy of DC vaccine clinical trials. Latent Membrane Protein-1 (LMP1) of Epstein-Barr virus was examined for its ability to mature and activate DC as a gene-based molecular adjuvant for DC vaccines. DC were transduced with an adenovirus 5 vector (Ad5) expressing LMP1 under the control of a Tet-inducible promoter. Ad5-LMP1 was found to mature and activate both human and mouse DC. LMP1 enhanced in vitro migration of DC toward CCL19, as well as in vivo migration of DC to the inguinal lymph nodes of mice following intradermal injection. LMP1-transduced DC increased T cell proliferation in a Pmel-1 adoptive transfer model and enhanced survival in B16-F10 melanoma models. LMP1-DC also enhanced protection in a vaccinia-Gag viral challenge assay. LMP1 induced high levels of IL-12p70 secretion in mouse DC when compared to standard maturation protocols. Importantly, LMP1-transduced human DC retained the capacity to secrete IL-12p70 and TNF in response to DC restimulation. In contrast, DC matured with Monocyte Conditioned Media-Mimic cocktail (Mimic) were impaired in IL-12p70 secretion following restimulation. Overall, LMP1 matured and activated DC, induced migration to the lymph node, and generated high levels of IL-12p70 in a murine model. We propose LMP1 as a promising molecular adjuvant for DC vaccines.

## Introduction

Dendritic cells (DC) are professional antigen presenting cells that play a central role in the adaptive immune response. A small number of DC can induce a robust immune response [1, 2], making ex vivo DC an attractive reagent for cancer immunotherapy [3]. However, DC immunotherapy clinical trials have shown limited efficacy to date against both cancer and HIV [2, 4–10]. The limited efficacy of current DC immunotherapy protocols may be related to weak or dysfunctional DC activation and maturation [9, 11]. In the absence of optimal activation, DC are unable provide T cell costimulation or cytokine-mediated T cell activation, two of the three signals necessary to induce a robust adaptive immune response [11, 12]. Indeed, suboptimal activation of DC can induce immune tolerance [11].

The cytokine cocktail mix Mimic, a combination of IL-1β, IL-6, TNF-α, and PGE2, is a commonly used reagent in DC immunotherapy trials. Mimic is used to mature monocyte-derived DC following antigen loading. The cytokine component of Mimic matures and activates DC. In contrast, the chemical PGE2, which improves migration of DC to the lymph node [13, 14], leads to DC dysfunction and exhaustion. For example, PGE2 induces a high IL-10/IL-12p70 ratio, Th2 polarization, and inhibits the secretion of IL-12p70 by DC following restimulation [15–18].

Latent Membrane Protein-1 (LMP1) is an Epstein-Barr virus (EBV) protein involved in the constitutive activation of infected B cells [19, 20]. LMP1 contains a transmembrane domain and an intracellular domain. The transmembrane domain aggregates LMP1 on the cell membrane. Aggregation of the transmembrane domain leads to signaling via TRAF molecules that interact with the LMP1 intracellular domain. This LMP1 TRAF mediated activation mimics signaling by the receptor CD40 [21], but in a ligand-independent manner. We therefore hypothesized that, based on the critical role of CD40 signaling on DC activation, LMP1 would be effective as a DC immunotherapy molecular adjuvant. We have previously evaluated the ability of LMP1 to increase DC maturation and activation when encoded within recombinant HIV-1 and SIV viruses [22, 23]. In this report, we investigated the ability of LMP1 to act as a replacement for Mimic in DC immunotherapy models. We chose to explore the use of adenoviral vector delivery of LMP1 based on previous work by others using adenoviral delivery of cancer antigens to DC [24, 25]. LMP1 activated and matured DC at levels equal or superior to Mimic. Importantly, LMP1 induced robust DC migration without the requirement for PGE2. LMP1 also increased the secretion of IL-12p70 following DC restimulation. Finally, LMP1 enhanced T cell responses and increased survival in murine DC therapeutic vaccine models for cancer and infectious disease. These data highlight the promise of LMP1 as an alternative to PGE2 for the induction of DC migration, and as a gene-based molecular adjuvant for DC immunotherapy.

## Materials and methods

### Production of recombinant adenovirus

Replication defective adenovirus (pAdEasy-1) was constructed containing codon-optimized Gag or GFP as an irrelevant antigen control, as described in manufacturer’s instructions (AdEasy Adenoviral vector system, Agilent tech). Genes were PCR amplified and cloned into the pAdenoVator-CMV5 shuttle vector (Qbiogene). The vectors were then electroporated into BJ5183 cells containing the pAdEasy-1 plasmid where homologous recombination occurred. After clonal selection, recombined vectors were linearized and transfected into AD293 cells (Stratagene). Adenovirus expressing LMP1 was constructed using the Adeno-X Tet-On 3G inducible system (Clontech). LMP1 (Genbank HQ706129.1) was cloned together with an IRES-GFP sequence to allow tracking of LMP1 expression by GFP fluorescence. LMP1-IRES-GFP was cloned into the Adeno-X system as described by the manufacturer’s instructions. Following sequencing to confirm the correct gene sequence, the viral vector was linearized and transfected into AD293 cells (Stratagene). Viruses were propagated in AD293 cells, then purified and concentrated using the Adeno-X Mega purification kit (Clontech). To determine infectious colony forming units (CFU), all viruses were titered using the Adeno-X Rapid titer kit (Clontech).

### Western blot to confirm protein production

A total of 1x10^6^ 293T cells were transduced with 1x10^6^ CFU of each viral construct in the presence of 1ug/ml doxycyline. After 48 hours, cells were harvested and lysed in RIPA buffer (Biorad) for Western blot. Proteins were denatured in 2% SDS with 1% DTT before loading on a 4–15% gradient Tris Glycine-SDS poly- acrylamide gels (Bio-Rad), electrophoresed, and blotted onto PVDF membrane (Pierce). The membrane was blocked using 5% (w/v) dry milk and then probed with Mouse anti-EBV LMP-1 antibody (Santa Cruz Biotechnology), followed by incubation with Peroxidase-Conjugated Donkey Anti-mouse IgG (Jackson Immunoresearch). The protein band was developed onto X-ray film using ECL detection reagent (Amersham).

### RT-PCR analysis of LMP1 mRNA

Quantitative RT-PCR was performed to measure LMP1 mRNA levels in transduced AD293 cells. A total of 1x10^6^ AD293 cells were transduced with 1x10^6^ CFU Ad5-LMP1 with or without 1ug/ml doxycycline added to the culture media at the time of transduction. AD293 cells were transfected with pcDNA3.1-LMP1 plasmid as a positive control using Lipofectamine 2000 (Invitrogen) according to manufacturer’s instructions. After 48 hours, total RNA was prepared from cells using the RNeasy kit (Qiagen), and reverse transcribed in a 20 μl reaction containing 0.1 μg of total RNA, 0.1 μg of oligo(dT), 200 U of reverse transcriptase (Finnzymes) and 0.2 μM each of dATP, dCTP, dGTP and dTTP. After 1 hr incubation at 40°C, cDNA products were generated. Real-time PCR then was performed using the Power SYBR Green Supermix (Applied Biosystems) with primers specific to LMP1. For normalization, GAPDH and β-actin real-time PCR was carried out on the same samples. Normalized mRNA levels for each transcript were calculated as (1/2ΔCt × 1,000), where ΔCt value = Ct (test mRNA)—Ct (GAPDH mRNA). To control for contamination with genomic DNA, parallel amplifications were performed in the absence of reverse transcriptase. These were uniformly negative.

### Luciferase reporter assay

NF-κB and IFN-β induction was measured using the dual-luciferase reporter assay system (Promega) according to manufacturer’s instructions. 293T cells were seeded on 24-well plates and transiently transfected with 30 ng of either NF-κB or IFN-β firefly luciferase reporter plasmid together with 4 ng pRL-TK and 300 ng of the various expression plasmids. Plasmids pcDNA3.1-FLAG-TRAF6 and pcDNA3.1-ΔRIG-I were transfected as positive controls. Empty vector pcDNA3.1 was used as a negative control. 36-48h later, luciferase activity was measured in the total cell lysate.

### SEAP assay

A NF-κB reporter cell line (293-SEAP) was used to monitor NF-κB activation by LMP1. These HEK293-derived cells express the gene for secreted embryonic alkaline phosphatase (SEAP) under the control of an NF-κB promoter [26]. 80,000 293-SEAP reporter cells, grown in DMEM medium with 10% FBS, were plated in each well of a 96-well plate. Viral stocks of Ad5-LMP1 were serially diluted and added to the reporter cells in triplicate, in the presence or absence of 1ug/ml doxycycline (Alfa Aesar) added at the time of transduction. After 36 h, 10 μl/well of the supernatants was added to the wells of a 96-well assay plate together with 100 μl/well of QUANTI-Blue Alkaline Phosphatase substrate (InvivoGen). The plates were incubated for 20 min at 20°C and OD was read at 650 nm.

### Mice

Female C57BL/6, Pmel-1 transgenic, or BALB/c mice (7–8 weeks old) were used in all experiments. Animals were housed at the University of Miami under the guidelines of the National Institutes of Health (NIH, Bethesda, MD). All animal experiments were performed in accordance with national and institutional guidance for animal care and were approved by the IACUC of the University of Miami. Animals were maintained under standard laboratory conditions (21°C, 12-hour light-dark cycle, lights on at 6 AM) with free access to standard chow and water. Mice were housed in individually ventilated plastic cages (groups of 2–5 mice/cage). Anesthesia was provided by ketamine/xylazine injection. Mice were sacrificed by CO2 inhalation. To minimize suffering of animals, animals were euthanized upon signs of pain. Criteria to determine when animals should be euthanized prior to end of study included signs of distress, open lesions or a tumor size of greater than 15 x 15 mm. Animals were monitored daily and euthanized immediately when criteria were met. All research staff were trained in the handling and care of mice by facility staff.

### Generation of BMDC

Bone marrow-derived murine DC (BMDC) were generated by standard methods [27] with the following modifications: Bone marrow cells were obtained from C57BL/6 mice and washed in RPMI 1640 media. The cells were then placed in non-tissue culture treated T75 flasks at a concentration of 1 x 10^6^ cells per ml in 20 ml complete RPMI (RPMI 1640 with 10% FBS, 20 μg/ml gentamycin sulfate, 50 μM 2-mercaptoethanol) containing 20 ng/ml murine recombinant GM-CSF and 10 ng/ml murine recombinant IL-4 (Peprotech, Rocky Hill, NJ)). Cells were cultured at 37°C, 5% CO2. On day 3, media was replaced with fresh complete RPMI containing cytokines. On day 5, cells were harvested, washed and resuspended in complete RPMI at 5 x 10^5^ cells/ml.

### BMDC maturation and activation

BMDC were generated from C57BL/6 mice as described above. 1x10^6^ DC were plated in each well of 6-well tissue culture-treated plates in a volume of 800ul. DC were transduced with Ad5-LMP1 or Ad5-GFP control (MOI = 50). DC were incubated with virus at 4°C for 1 h, followed by 3 h at 37°C. Complete media was then added to 2ml with a final concentration of 1ug/ml doxycyline. As a positive control, cytokine mix Mimic (5 ng/ml TNF-α (Peprotech), 5ng/ml IL-1b (Peprotech), 750ng/ml IL-6 (Peprotech), and 1ug/ml PGE2 (Sigma)) was used to mature DC. Cells were incubated for 36 hours at 37°C, harvested, and stained with the following antibodies: anti-mouse CD80 clone 16-10A1, CD86 clone GL1, CD40 clone 1C10, CD83 clone Michel-17, MHC Class II (I-A/I-E) clone M5-114.15.2, and CCR7 clone 4B12 (all from eBioscience). Tubes were also stained with hamster anti-mouse CD11c clone N418 PE-Cyanine7 conjugate (eBioscience) to allow for gating on CD11c+ DC. After flow cytometry analysis, the mean fluorescence intensity (MFI) for each antibody was calculated for CD11c+ dendritic cells under each experimental condition. FlowJo 7.6.4 flow cytometry analysis software (FlowJo, Ashland, OR) was used for analysis. Three independent wells were analyzed for each condition.

### Transduction of BMDC and MDDC

DC were removed from their flasks by placing in the fridge for 1 hour before gentle pipetting to isolate unattached and loosely attached cells. 1.0x10^6^ DC were plated in a 6 well plate (800ul plain RPMI-1640). DC were transduced with Ad5-GFP, Ad5-Gag, Ad5-gp100 or Ad5-LMP1 at the indicated MOI. DC were first cultured in the fridge for 1 hour, then incubated at 37°C for 3 hours. 2 ml fresh DC media including 1ug/ml doxycycline was then added for overnight culture.

### Human and mouse CBA

Mouse BMDC and human MDDC culture supernatants were collected 12, 24, and/or 36 hours following transduction. Supernatants were analyzed using the Mouse Inflammatory Cytokine CBA kit or the Human Inflammatory Cytokine CBA kit (BD Bioscience) according to manufacturer’s protocols and run on an LSRFortessa. Cytokine values were calculated and represented as pg/ML. Note that Mimic cytokines were not removed from the culture, affecting levels of IL-6 and TNF-α for the Mimic sample.

### In vivo migration

BMDC in vivo migration was performed as previously described [28] with the following modifications. BMDC were generated from C57BL/6 mice as described above. BMDC were then transduced at an MOI of 50 with Ad5-LMP1, Ad5-Gag negative control, or Ad5-Gag DC matured with Mimic. After 36 hours, DC were CFSE labeled and injected intradermally into the flank of C57BL/6 mice. After 48 hours, the inguinal lymph nodes were dissected and processed into single cell suspensions. Cells were stained with hamster anti-mouse CD11c clone N418 PE-Cyanine7 conjugate (eBioscience) and analyzed by flow cytometry. The total number of migrated CD11c+CFSE+ dendritic cells was calculated based upon volume.

### PMEL assay

Pmel-1 transgenic (Thy1.1+Vβ13+) mice were bred in house. BMDC were generated from C57BL/6 mice as described above. On day 3, DC were transduced at an MOI of 50 with Ad5-gp100. On day 4, DC were with transduced at an MOI of 50 with Ad5-GFP, Ad5-GFP + Mimic maturation cocktail, or Ad5-LMP1 in the presence of 1ug/ml doxycycline. On day 5, 1x10^6^ DC were injected intradermally into the flank of C57BL/6 mice that had been adoptively transferred with 1x10^6^ purified CD8+ T cell isolated from a Pmel-1 mouse spleens 24 hours previously by positive bead selection (Miltenyi). For a positive control, LPS (Sigma Aldrich) + gp100 peptide (KVPRNQDWL) (American Peptide) were injected subcutaneously into C57BL/6 mice. After 5 days, spleens were harvested and processed for single cell suspensions. Spleen preps were stained with anti-mouse CD3e clone 500A2, anti-mouse CD8a PerCP clone 53–6.7, anti-mouse Thy-1.1 clone OX-7 (BD Bioscience). Data represented as percent of CD3+ CD8+ T cells that were Thy1.1+.

### Tumor DC therapy model

C57BL/6 mice (7–8 week old) were injected either IV or intradermally in the flank with 50,000 B16-F10 melanoma cells. After 3 days, mice were vaccinated with 1x10^6^ DC intradermally every 3 days for a total of 6 vaccinations. DC were either transduced with Ad5-gp100 and Ad5-GFP, Ad5-gp100 and Ad5-LMP1, or Ad5-gp100 and Ad5-GFP matured with Mimic cytokine cocktail. As a positive control, GM-CSF expressing B16/F10 tumor cells (GVAX) were irradiated (5,000 rad) and 1 × 10^6^ cells were injected subcutaneously at the same vaccination schedule as DC. Doxycycline (2mg/ml) was added to the mouse water bottles from the time of injection until the termination of the experiment to ensure continued expression of the Tet-inducible Ad5-LMP1. Tumors area was measured every 2 days for flank tumors and mice were euthanized when tumors reached 15x15mm. For the IV lung metastases model, mice were sacrificed 21 days after challenge and lungs harvested.

### Vaccinia viral challenge

7–8 week-old female Balb/C mice were immunized (nine mice per group in two independent experiments) with 100,000 dendritic cells transduced with Ad5-Gag and Ad5-GFP, Ad5-Gag and Ad5-LMP1, or Ad5-Gag and matured with Mimic cytokine cocktail. One month after immunization, mice were challenged i.p with 1x10^7 vp vaccinia-Gag virus. Five days following challenge, mice were sacrificed and both ovaries and fallopian tubes were removed and homogenized in 500ul PBS. For measurement of virus titers, samples were sonicated, and evaluated in triplicate by 10-fold serial dilution on CV-1 cells plated in 24 well plates. After 48 hour incubation, plates were stained with 0.1% (w/v) crystal violet in 20% ethanol. Plaques were counted to determine PFU of virus.

### Monocyte-derived dendritic cell preparation

For HIV infected patient samples, patients were recruited from the University of Miami AIDS Clinical Research Unit. The study was approved by the University of Miami Institutional Review Board. All patients signed informed consent in accordance with the University of Miami Institutional Review Board. Whole blood from buffy coat or from HIV+ patients was centrifuged over ficoll (GE Healthcare) at 1,000 x g for 25 min at room temperature. The monocyte layer was collected, diluted in PBS, and centrifuged sequentially at 1800 rpm for 10min, 1100rpm for 10min, then 1000rpm for 10 min with aspiration and resuspension between each spin. PBMC were then resuspended in complete media: RPMI 1640 (Hyclone) containing 5% human AB serum (Male only) (Lonza), PenStrep (1x), and L-glutamine. To isolate monocytes, 8 × 10^7^ HIV patient PBMC were placed in a T75 flask or 2x10^8^ buffy coat PBMC were placed in a T175 flask in complete media and cultured for 2 hours at 37°C in 5% CO2. Unattached cells were removed by washing with warm media three times. Unattached cells (defined as peripheral blood lymphocytes, PBL) were pelleted, resuspended in 80% FBS + 10% Glucose + 10% DMSO, and frozen. The adherent monocyte cells were cultured in 20 ml (T75) or 40 ml (T175) of media containing 500U/ml IL-4 (R&D systems) and 1000U/ml GM-CSF (BERLEX Inc). After 5 days of culture, flasks containing immature DC were placed at 4°C for 30 minutes to detach DC, then collected by gentle pipetting to isolate unattached and loosely attached cells. Any remaining adherent cells were discarded.

### MDDC maturation and activation assay

MDDC were matured and then stained for markers of maturation and activation (anti-human CD14 clone M5E2, CD86 clone 2331 (FUN-1), CD80 clone L307.4, HLA-DR clone TU36, CD83 clone HB15e, CD40 clone 5C3, CD197 clone 3D12, and CD11c clone 3.9) (BD Bioscience).

### In vitro migration assay

Day 5 DC were removed from their flasks and transduced as described with Ad5-GFP or Ad5-LMP1 at an MOI of 100 or 500. Following 36 hours of culture in the presence of Doxycycline (1ug/ml) or Mimic cytokine cocktail, 1.5x10^5^ matured dendritic cells were added to 8 μm transwells (Greiner Bio-one) in triplicate. Transwells were held in a 24 well plate in media containing 150 ng/mL of CCL19 (Peprotech). After a 90-minute incubation, transwells were removed and DC in the lower chamber were removed with EDTA and counted.

### DC:T cell co-culture and ELISPOT assay

MDDC generated from PBMC preps from HIV patients were transduced with Ad5-Gag and matured with Ad5-LMP1, Mimic cytokine mix, or Ad5-GFP control. Following overnight culture, DC were washed and mixed with 1x10^5^ autologous peripheral blood lymphocytes at a 1:10 DC:T cell ratio and cultured for 12 days. Following culture, IFN-γ and IL-2 ELISPOT assays were performed on lymphocyte cultures to determine antigen specific cytokine secretion. ELISPOT assays were carried out per the manufacturer’s instructions (R&D Systems) using 96-well MAIP plates (Millipore). 1×10^5^ cells/well were added to each well of the plate, and stimulated for 18 h at 37°C, 5% CO2, in the presence of HIV-1 Gag peptide pool (5 ug/ml). An OVA peptide (negative control) and PMA/Ionomycin (positive control) were also included to calculate the number of antigen-specific ELISPOTs. After 18 h, spots were developed with AEC substrate kit (BD Bioscience), according to manufacturer’s instructions. The membrane was read by automated reader (CTL Immunospot) for quantitative analyses of the number of IFN-γ or IL-2 spot forming cells (SFC) per million cells plated, subtracting negative control values.

### Flow cytometry analysis

Flow cytometric data was analyzed using FlowJo7.6.4.

### Statistical analysis

Graph pad Prism 6.0 software was used to calculate significance using a one-way ANOVA, followed by either a two-tailed Student's *t*-test or a Mann-Whitney *t*-test as noted. A log-rank test was used to determine the significance of the differences between groups in Kaplan-Meier survival plots. A p value of 0.05 was considered significant. In all figures, *p* values are labeled by asterisks denoting *p* <0.05 (*), *p* <0.01 (**), and *p* <0.001 (***).

## Results

### Construction of adenovirus vector expressing LMP1

**Fig 1:**
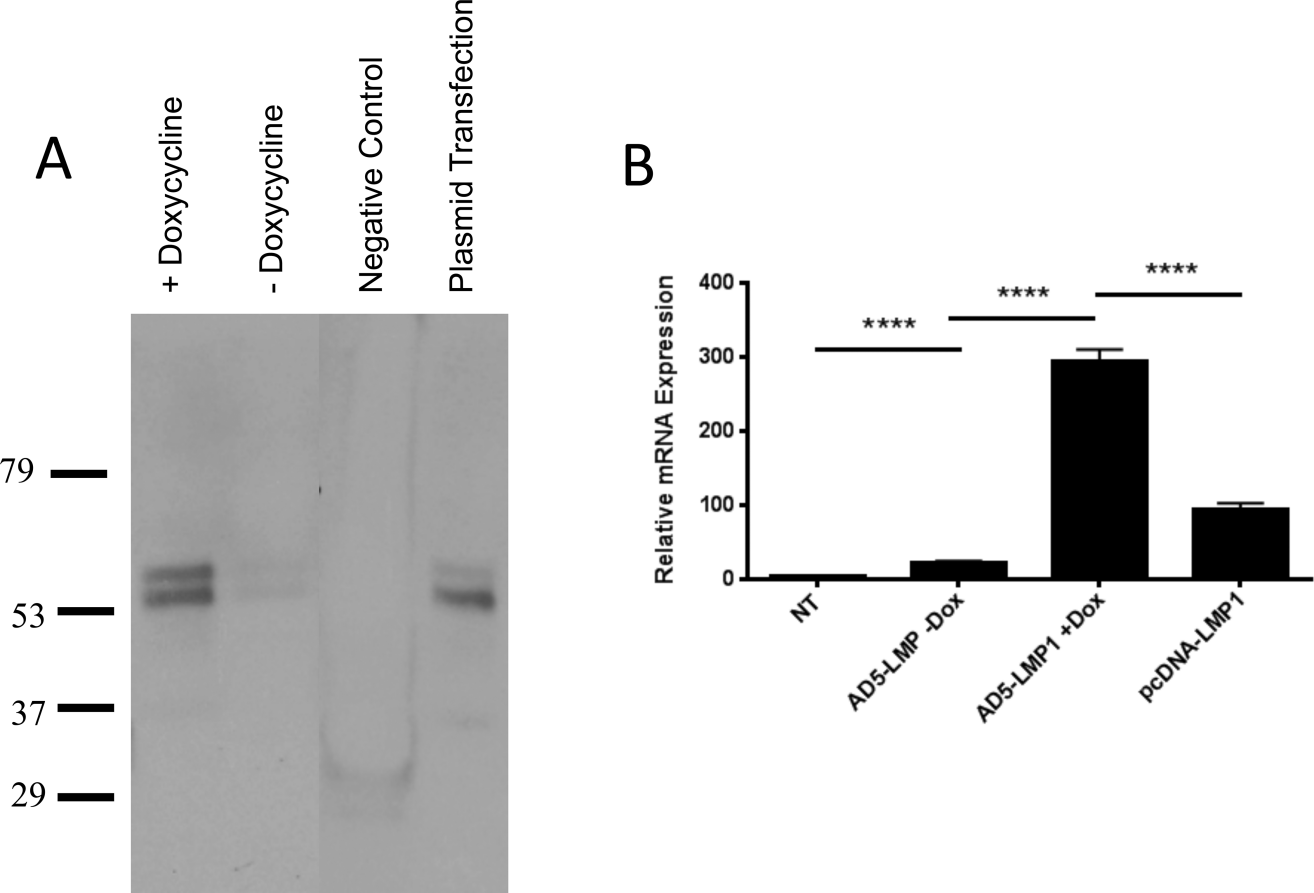
Construction of Ad5-Tet-On-LMP1. AD293 cells were transduced with Ad5- LMP1 in the presence or absence of doxycycline. Cells were transfected with plasmid pcDNA3.1-LMP1 as a positive control. **(A)** After 48 hours, cells were harvested and lysed in RIPA buffer. Proteins were denatured and run on a 4–15% gel. Membrane was probed with mouse Anti-EBV LMP1 antibody. **(B)** After 48 hours, RNA was purified and reverse transcribed for real-time PCR analysis. AD293 cells were transfected with pcDNA3.1-LMP1 plasmid as a positive control. Real-time PCR was performed with SYBR Green using LMP1 specific primers. For normalization, GAPDH and β-actin real-time PCR was carried out on the same samples.

LMP1 was cloned into a replication-defective Ad5 viral vector system for transduction of DC. Constructs were cloned such that LMP1 expression was directly linked to GFP expression using an internal ribosomal entry site (IRES). The Ad5 expression system was tetracycline-inducible, allowing for tight control of LMP1 expression and the ability to evaluate transduced cells both before and after LMP1 expression. When doxycycline (Dox) was added to culture media, LMP1 expression was detectible by Western blot (Fig 1A). In the absence of doxycycline, only a faint LMP1 band was observed. To further evaluate expression by the tetracycline inducible system, real-time PCR was performed on AD293 cells transduced with Ad5-LMP1 in the presence or absence of doxycycline (Fig 1B). Transfection with plasmid pcDNA3.1-LMP1 was used as a positive control. Doxycycline induced high levels of LMP1 expression compared to the Dox- group, three times higher than plasmid-transfected control (p = 0.0001).

### LMP1 increased NF-κB induction and IFN-β expression

**Fig 2:**
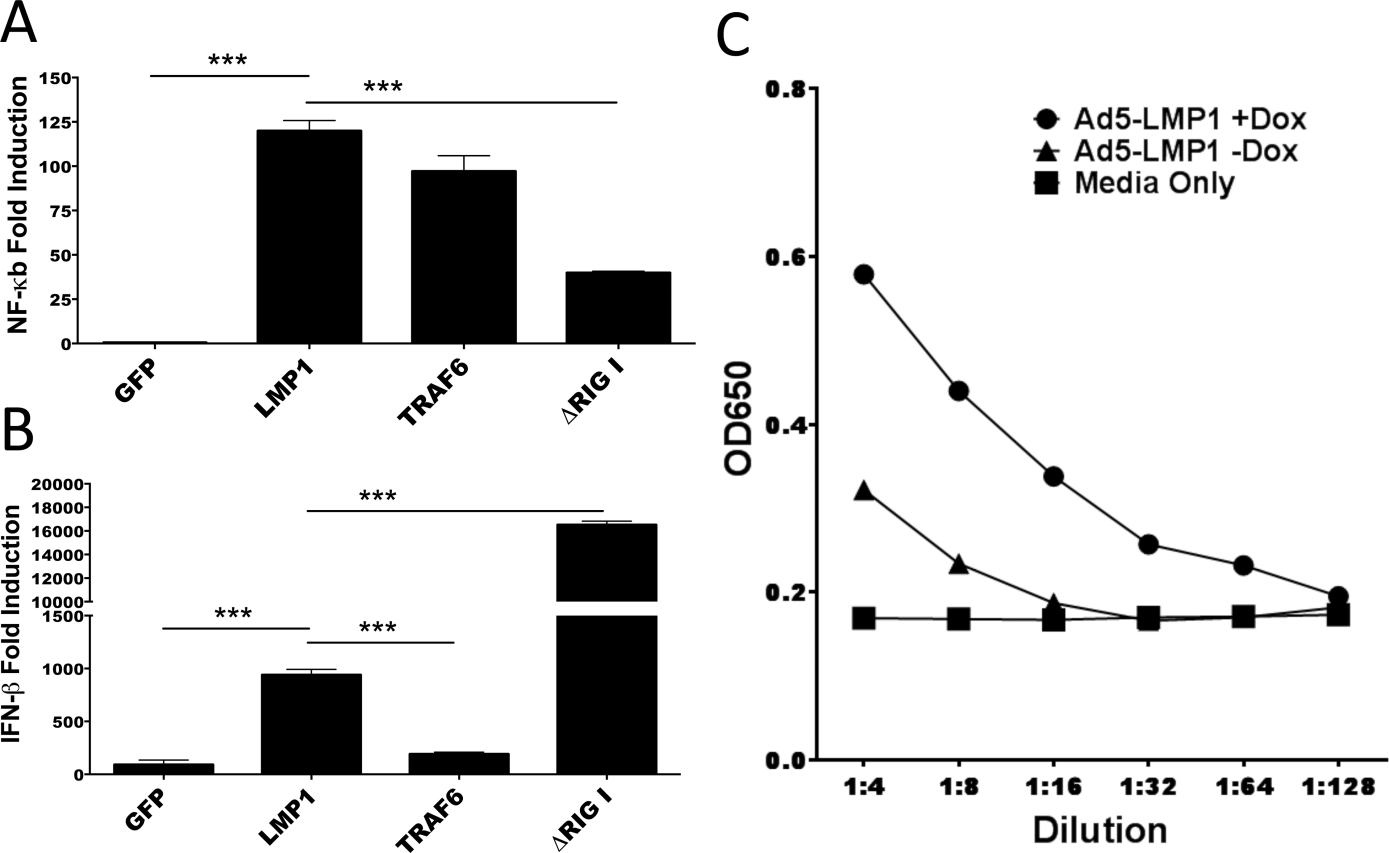
Biological activity of Ad5-LMP1. **(A, B)** 293T cells were transfected with either NF-κB **(A)** or IFN-β **(B)** firefly luciferase reporter plasmid together with pRL-TK and either LMP1 or control expression plasmids. As positive controls, pcDNA3.1-FLAG-TRAF6 and pcDNA3.1-ΔRIG-I were used. 36-48h later, luciferase activity was measured in total cell lysate. **(C)** Viral stocks of Ad5-LMP1 were serially diluted and added to 293-SEAP reporter cells in triplicate, in the presence or absence of doxycycline. After 36h, secreted alkaline phosphatase was measured. Data represent typical results from three independent experiments.

To test the biological activity of LMP1, 293T cells were transfected with pcDNA3.1-LMP1 together with NF-κB or IFN-β luciferase reporter plasmids. TRAF6 and ΔRIGI were used as NF-κB and IFN-β positive controls, respectively. LMP1 induced NF-κB levels >100-fold higher than the GFP control (p<0.0001) (Fig 2A). LMP1 also induced IFN-β levels >500-fold higher than GFP (p = 0.0003) (Fig 2B). LMP1 induced similar levels of NF-κB induction compared to the TRAF6 control, but significantly lower levels of IFN-β induction compared to the ΔRIG-I control. Despite high levels of NF-κB induction, we did not observe signs of toxicity in any of the cells transduced, including 293 cells and mouse and human DC.

To confirm biological activity of Ad5-LMP1, an NF-κB inducible SEAP reporter HEK293 cell line was used. In the presence of doxycycline, Ad5-LMP1 induced SEAP secretion at all supernatant dilutions (Fig 2C). In the absence of doxycycline, only low levels of SEAP activity were observed, consistent with low background levels of LMP1 expression (Fig 1A).

### Ad5-LMP1 activated and matured mouse bone marrow-derived dendritic cells (BMDC)

**Fig 3:**
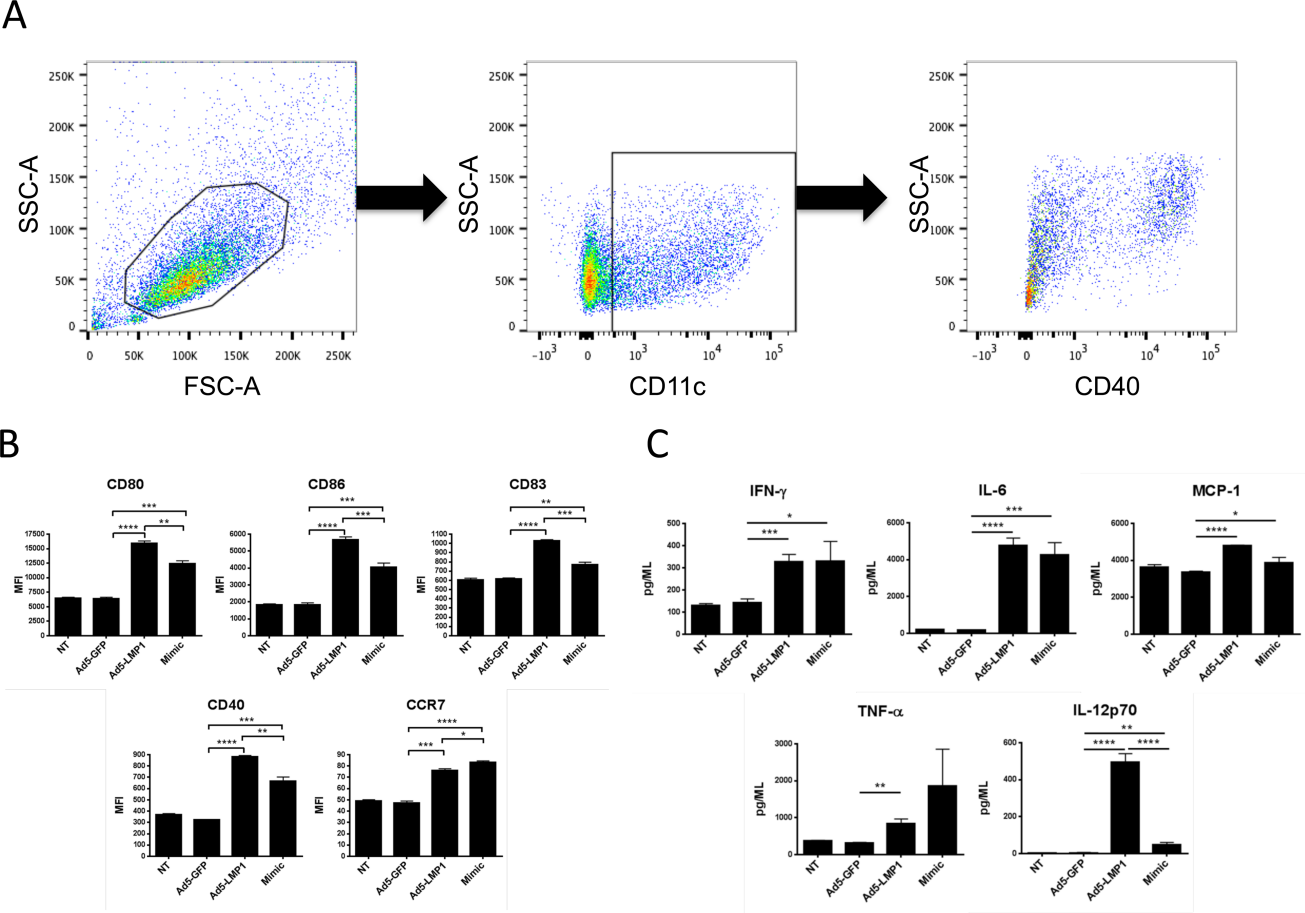
Ad5-LMP1 enhanced bone marrow-derived dendritic cell maturation and cytokine secretion. BMDC were transduced with Ad5-LMP1 or control virus in the presence of doxycycline. Mimic was added as a positive control. **(A)** Gating strategy used to define CD11c+ DC from BMDC cultures. **(B)** On day 5, cells were stained for maturation markers, and analyzed by flow cytometry. **(C)** On day 5, supernatant was collected and analyzed using the BD mouse inflammation CBA kit. Note that Mimic cytokines were not removed from the Mimic control culture before analysis, and IL-6 and TNF-α levels may represent exogenous protein. Data represent results from three independent experiments.

Mouse BMDC were transduced with Ad5-LMP1 or controls and 36 hours later cells were analyzed for markers of maturation and activation by flow cytometry. DC were prepared fresh for each administration. DC transduced with Ad5-LMP1 showed significantly higher levels of activation and maturation compared to Mimic or Ad5-GFP controls. The gating strategy is shown in Fig 3A. CD80, CD86, CD40, and CD83 were significantly upregulated by Ad5-LMP1 (Fig 3B), consistent with the ability of LMP1 to mimic constitutive CD40-mediated signaling [20]. Ad5-LMP1 also induced significantly higher CCR7 expression compared to the Ad5-GFP (p = 0.0002), but significantly lower levels of CCR7 compared to Mimic (p = 0.014) (Fig 3B).

### LMP1-transduced mouse BMDC expressed high levels of proinflammatory cytokines

Next, we evaluated the ability of LMP1 to enhance DC proinflammatory cytokine secretion. LMP1-DC secreted significantly higher levels of IL-12p70, TNF, IFN-γ, MCP-1, and IL-6 compared to GFP-DC (Fig 3C). TNF, IFN-γ, MCP-1 and IL-6 cytokine levels were not significantly different between LMP1-DC and Mimic-DC. Importantly, LMP1-DC secreted significantly higher levels of IL-12p70 compared to Mimic-DC (p<0.0001).

### LMP1 enhanced BMDC in vivo migration without the requirement for PGE2

**Fig 4:**
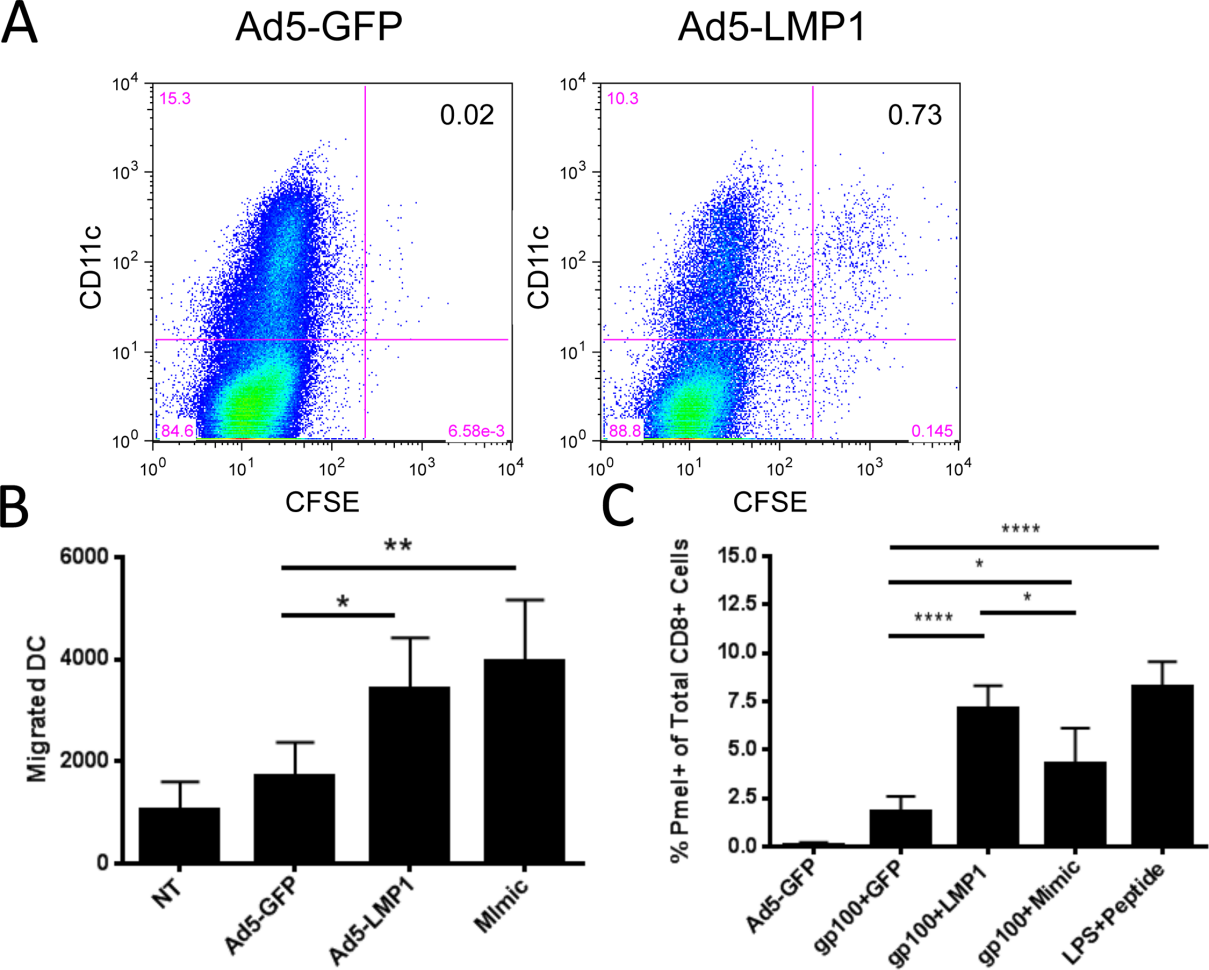
Ad5-LMP1 transduction enhanced mouse bone marrow-derived dendritic cell migration and T cell stimulation. **(A)** Bone marrow derived DC were transduced with Ad5-LMP1 or Ad5-GFP at an MOI of 50 on day 5. After 36 hours, DC were CFSE labeled and 5x10^5^ DC were injected intradermally into the flank of a C57BL/6 mouse. Mice were given doxycycline in drinking water from the time of injection. After 48 hours draining inguinal lymph nodes were processed for single cell suspension. Cells were stained for CD11c and run on flow. Migrated cells were defined as CFSE+ CD11c+. **(B)** BMDC were transduced with Ad5-LMP1 or Ad5-GFP. Mimic was added as a positive control. After 36 hours, DC were CFSE-labeled and 5x10^5^ DC injected intradermally into the flank of C57BL/6 mice (5 mice/group). After 48 hours, inguinal lymph nodes were isolated and processed for single cell suspension. Migrated DC were defined as CFSE+ CD11c+. **(C)** BMDC were transduced with Ad5-GP100. 24h later, DC were transduced with Ad5-GFP, Ad5-GFP plus Mimic cytokines, or Ad5-LMP1. The next day, 1x10^6^ DC were injected intradermally into the flank of C57BL/6 mice adoptively transferred 24h previously with 1x10^6^ CD8+ T cells isolated from a Pmel-1 transgenic mouse (5 mice/group). For positive control, LPS and gp100 peptide was injected subcutaneously. 5 days following DC injection, splenocytes were isolated and stained for CD3, CD8, and Thy-1.1. Data represented as percent CD3+ CD8+ T cells that were Thy1.1+. Data represent results from three independent experiments.

For DC to be effective therapeutic agents, they must possess the ability to migrate to the draining lymph node upon injection. To determine the migratory abilities of LMP1-DC, transduced DC were CFSE labeled and injected intradermally into the flank of C57BL/6 mice. After 48 hours, draining inguinal lymph nodes were removed and cells analyzed by flow cytometry for the total number of migrated CD11c+CFSE+ DC. CD11c+CFSE+ DC could be readily detected by flow cytometry (Fig 4A). Ad5-LMP1 significantly enhanced migration compared to the Ad5-GFP control (p = 0.0134) and LMP1-DC migration was similar to that induced by Mimic (Fig 4B). Approximately 0.6% of injected CFSE-labeled Ad5-LMP1 DC migrated to the proximal lymph node.

### LMP1-DC induced antigen-specific T-cell responses

Next, we evaluated whether LMP1 could enhance the ability of mouse DC to stimulate a T cell response. The Pmel-1 CD8+ T cell model was used. Pmel mice carry a rearranged T cell receptor transgene specific for human gp100 [29]. To evaluate Pmel responses, C57BL/6 mice were adoptively transferred with purified Pmel CD8+ T cells. One day later, DC transduced with Ad5-gp100 and either Ad5-LMP1, Ad5-GFP or Mimic were injected i.d. into the flank. After 5 days, spleens were analyzed for CD3+CD8+Thy1.1+ Pmel cells. As shown in Fig 4C, LMP1-DC stimulated a robust Pmel response, significantly higher than either GFP-DC (p<0.0001) or Mimic-DC (p = 0.0188), and equivalent to positive control mice vaccinated with LPS + gp100 peptide. PMEL cells were approximately 7.5% of circulating CD8+ T cells, suggesting that LMP1-matured DC were effective antigen presenting cells.

### LMP1-DC slowed B16-F10 tumor growth, delayed tumor onset, and increased survival

Next, we determined whether LMP1 could enhance a cancer DC immunotherapy model. C57BL/6 mice were challenged with B16-F10 tumors in the flank and monitored for tumor onset, growth, and survival. On day 3 following tumor challenge, animals were vaccinated with 1×10^6^ DC transduced with Ad5-gp100 antigen together with Ad5-LMP1, Ad5-GFP or Mimic. Mice were vaccinated every 3 days for a total of 6 injections. As a gold standard treatment, 1×10^6^ GVAX cells (B16/F10 tumor cells expressing GM-CSF) [30] were irradiated and given s.c. on the same vaccination schedule as DC immunotherapy.

**Fig 5:**
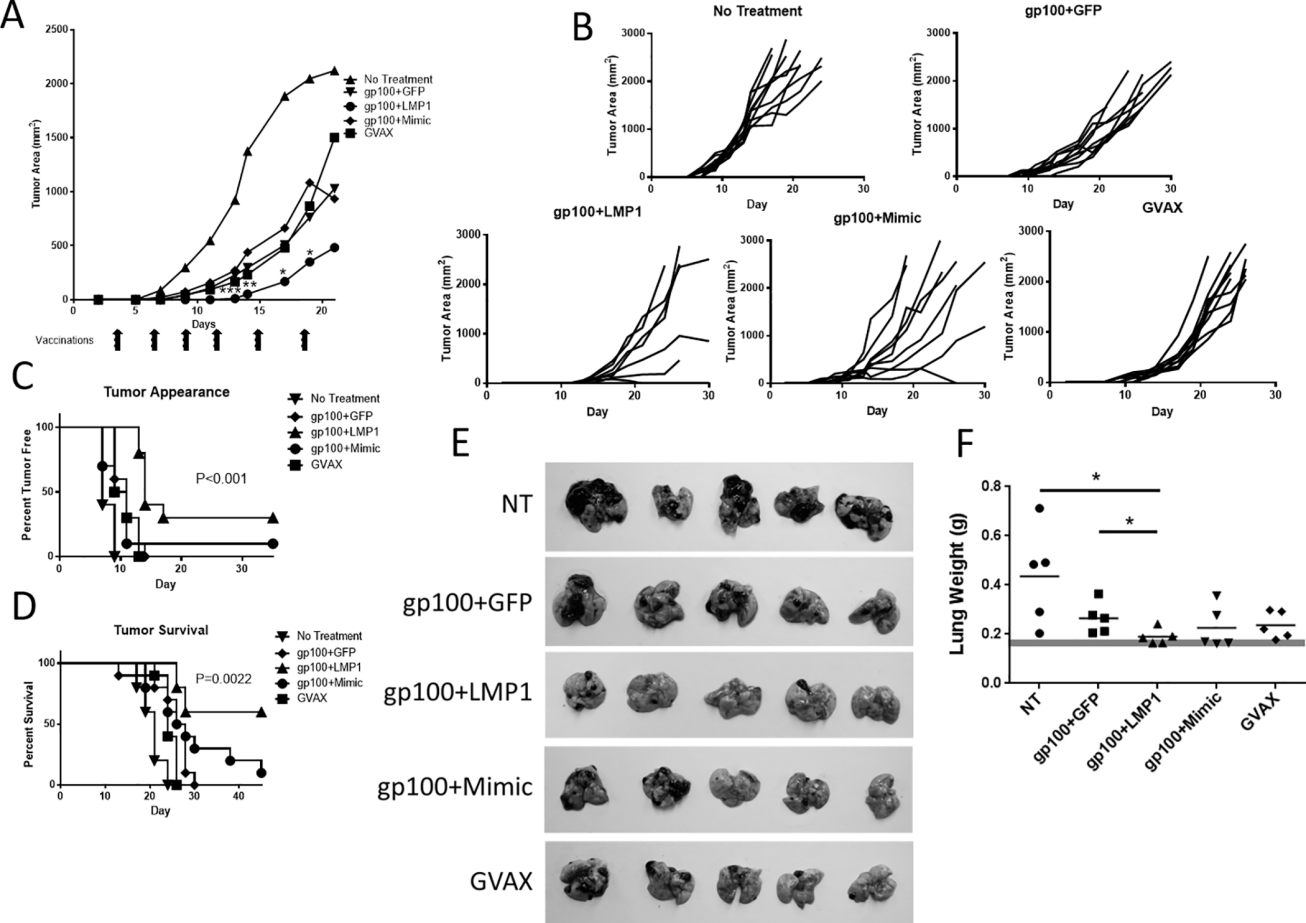
LMP1-DC enhanced responses against B16-F10 tumors. 5x10^4^ B16 tumor cells were injected either intradermally into the flank **(A-D)** or i.v. **(E,F)** into C57BL/6 mice. Three days later mice were treated with BMDC transduced with Ad5-GP100 antigen and matured with either Ad5-LMP1 or Mimic. Mice were given doxycycline in drinking water from the time of injection. Mice were vaccinated with 1x10^6^ DC injected intradermally into the flank every 3 days, starting 3 days after tumor challenge, for a total of 6 treatments. **(A)** Mean tumor area. Asterisks denote *p* <0.05 (*) or *p* <0.01 (**) comparing the gp100 + LMP1 group to all other groups (n = 10 mice per group, combined from two independent experiments). **(B)** Individual mouse tumor growth curves. **(C)** Tumor appearance survival curve. **(D)** Mouse survival curve. **(E)** Images of lung tumors at day 21 (n = 5 mice per group). **(F)** Lung weight.

Mice vaccinated with LMP1-DC showed a significant delay in tumor growth compared to all other treatment groups on days 13–19 (Fig 5A and 5B). LMP1 also significantly delayed tumor appearance compared to all other groups (Fig 5C). Palpable tumors did not appear until day 15 compared to day 7–10 with all other treatments. LMP1-DC significantly enhanced survival compared to GVAX (p = 0.0022) (Fig 5D). For 3/10 Ad5-LMP1 adjuvanted animals, tumors were not observed, and three additional animals generated a slow growing or regressing tumor that allowed survival to day 45.

### LMP1 reduced tumor burden in a lung metastases model

Next, LMP1 was tested in a lung metastases model to evaluate the ability of LMP1-DC to prevent establishment and/or growth of metastatic lesions. C57BL/6 mice were injected i.v. with 50,000 B16-F10 tumor cells. Animals were vaccinated 6x as outlined in Fig 5A, starting on day 3 following tumor challenge. As a gold standard, 1×10^6^ irradiated GVAX cells were injected s.c. following the same vaccination schedule. Lungs were harvested on day 21 following challenge. We observed fewer tumors and a significant reduction in lung weight for animals treated with LMP1-DC compared to no treatment (p = 0.025) or GFP-DC (p = 0.0453) (Fig 5E and 5F). GVAX and Mimic-DC were moderately effective at reducing tumor burden as measured by lung weight, but did not reach statistical significance compared to no treatment or DC given gp100+GFP (Fig 5F).

### Vaccination with LMP-DC enhanced protection from vaccinia-Gag challenge

**Fig 6:**
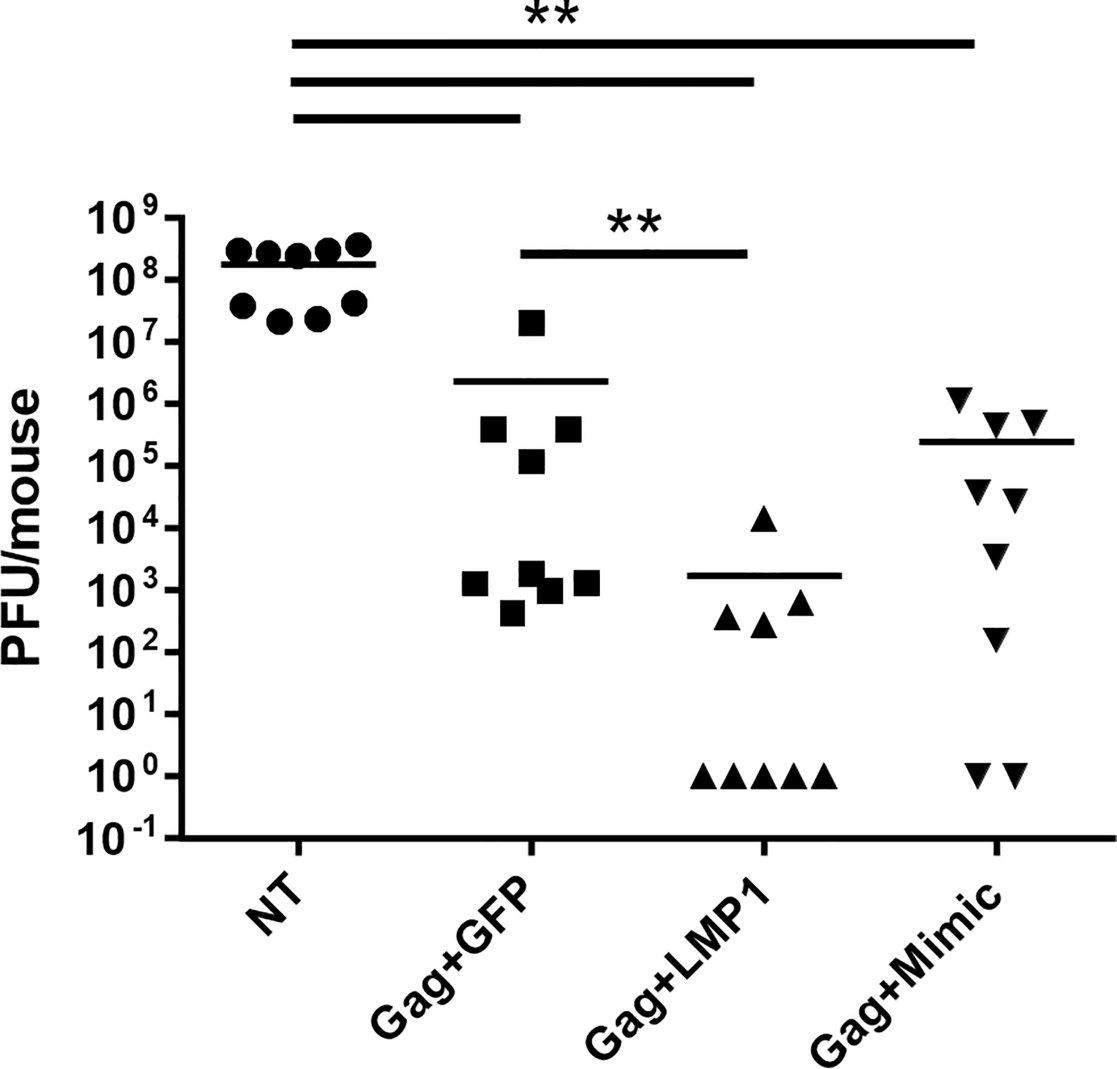
Ad5-LMP1 DC reduced viral titers of vaccinia-Gag in a prophylactic DC vaccination model. BMDC were transduced with Ad5-Gag and matured with either Ad5-LMP1 or Mimic in the presence of doxycycline. Mice were vaccinated with 1x10^5^ DC injected intradermally into the flank. Mice were given doxycycline in drinking water from the time of injection. 4 weeks post-vaccination, mice were challenged with 1x10^7^ viral particles vaccinia-Gag. Five days following challenge, ovaries were assayed for viral titer. Data combine the results from two independent experiments (n = 9 mice per group).

To further evaluate LMP1-DC in vivo, we tested LMP1-DC immunotherapy using a prophylactic vaccination and viral challenge model. Mice were vaccinated once with 1x10^5^ DC transduced with antigen (Ad5-Gag) and either Ad5-LMP1, Ad5-GFP or Mimic. Four weeks later, mice were challenged i.p. with vaccinia-Gag virus. Five days following viral challenge, ovaries were titered for vaccinia virus production. We observed a mean 5-log reduction in vaccinia titers in mice vaccinated with LMP1-DC compared to no treatment (p = 0.0013) (Fig 6). 5/9 mice vaccinated with LMP1-DC had undetectable viral titers, compared to 2/9 animals vaccinated with Mimic-DC. We also observed a statistically significant reduction in viral titers between GFP-DC and LMP1-DC (p = 0.0011).

### Ad5-LMP1 matured and activated human monocyte-derived dendritic cells

**Fig 7:**
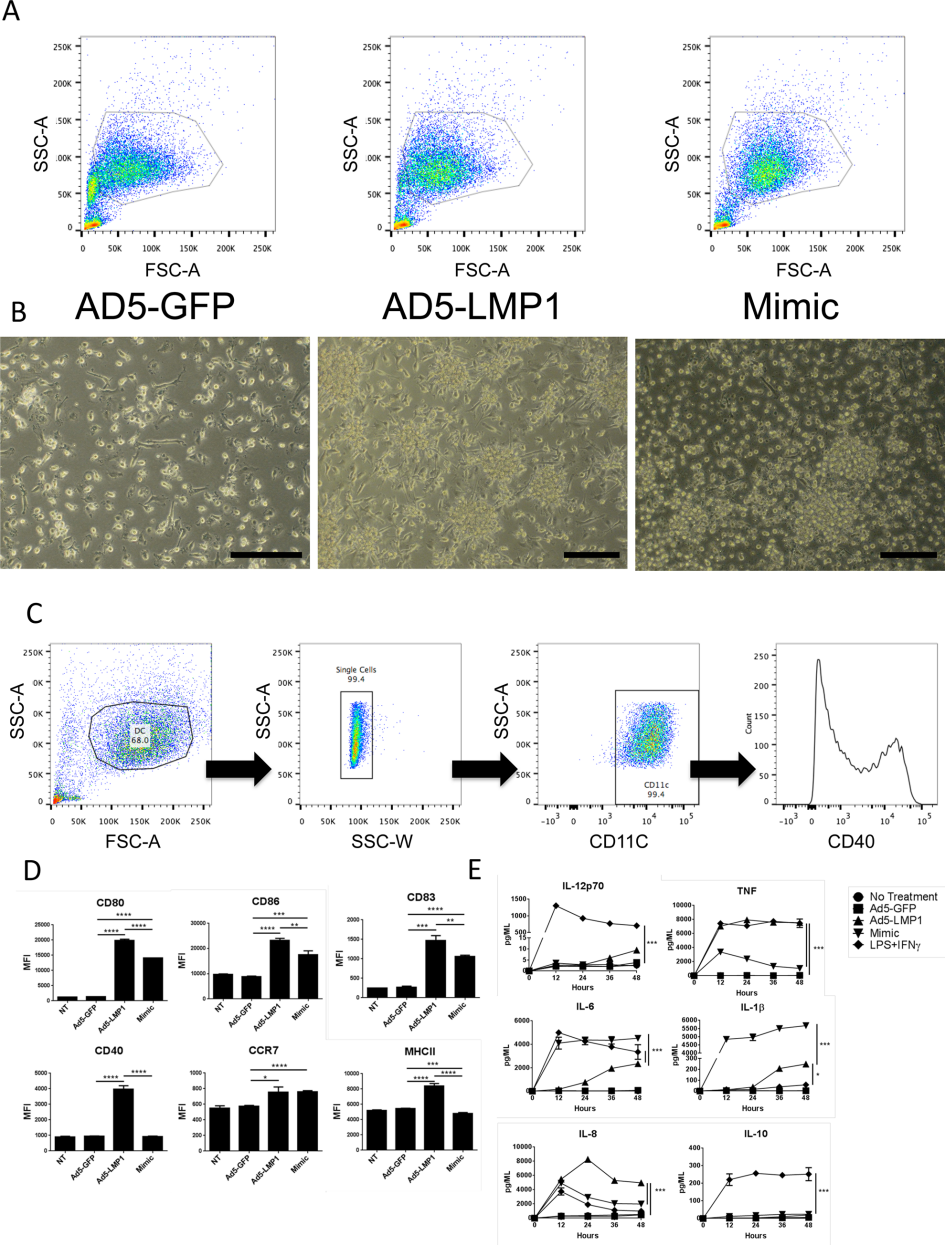
Ad5-LMP1 enhanced maturation, cytokine production of human monocyte-derived dendritic cells. Human monocytes were isolated from buffy coat and cultured in the presence of GM-CSF and IL-4 for 5 days to generate DC. On day 5, cells were infected with Ad5-LMP1 or Ad5-GFP at an MOI of 100 in the presence of doxycycline. Mimic cytokine maturation cocktail was added on day 5 as a positive control. **(A)** Cells were analyzed by flow cytometry and representative forward vs. side scatter plots are displayed. **(B)** Images of DC were taken on day 7 before harvesting for maturation staining. Scale bar equals 100μm. **(C)** Representative gating strategy used to determine maturation of MDDC. **(D)** DC were stained for maturation markers, and analyzed by flow cytometry. **(E)** Supernatant was collected every 12 hours for 2 days and cytokine measured by CBA. TNF-α, IL-6 and IL-1β levels for Mimic sample may represent residual protein from the Mimic cocktail. Data represent results from three independent experiments.

Based on promising mouse data, we evaluated Ad5-LMP1 transduction of human monocyte derived DC (MDDC). DC transduced with Ad5-LMP1 showed classic morphological signs of activation, including increased cell clumping and dendrite formation compared to Ad5-GFP transduced DC (Fig 7B). However, these morphological effects did not significantly alter forward and side scatter of the cells (Fig 7A), allowing flow cytometry analysis.

Next, we analyzed activation and maturation of human MDDC. The gating strategy is shown in Fig 7C. Typically >99% of MDDC were CD11c+. Following transduction with Ad5-LMP1, human LMP1-DC showed a significant upregulation of activation markers including CD80, CD86, CD83, CCR7, CD40, and MHCII (Fig 7D). With the exception of CCR7, human LMP1-DC showed significantly higher levels of maturation markers compared to Mimic-DC. Proinflammatory cytokine secretion was also significantly increased in human LMP1-DC compared to GFP-DC (Fig 7E). LMP1-DC cytokine secretion either peaked at 12–24 hours after transduction (TNF and IL-8), or gradually increased during the 48-hour culture (IL-6, IL-1β, and IL-12p70). LMP1-DC induced significantly higher levels of TNF and IL-8 compared to Mimic-DC. While human MDDC did not secrete high levels of IL-12p70 following transduction with Ad5-LMP1 (Fig 7E), we did observe a modest increase in IL-12p70 secretion at 48h for LMP1-DC compared to Mimic-DC. As a positive control, DC stimulated with LPS + IFN-γ induced high levels of most cytokines, including IL-12p70 and IL-10, but not IL-1β.

**Fig 8:**
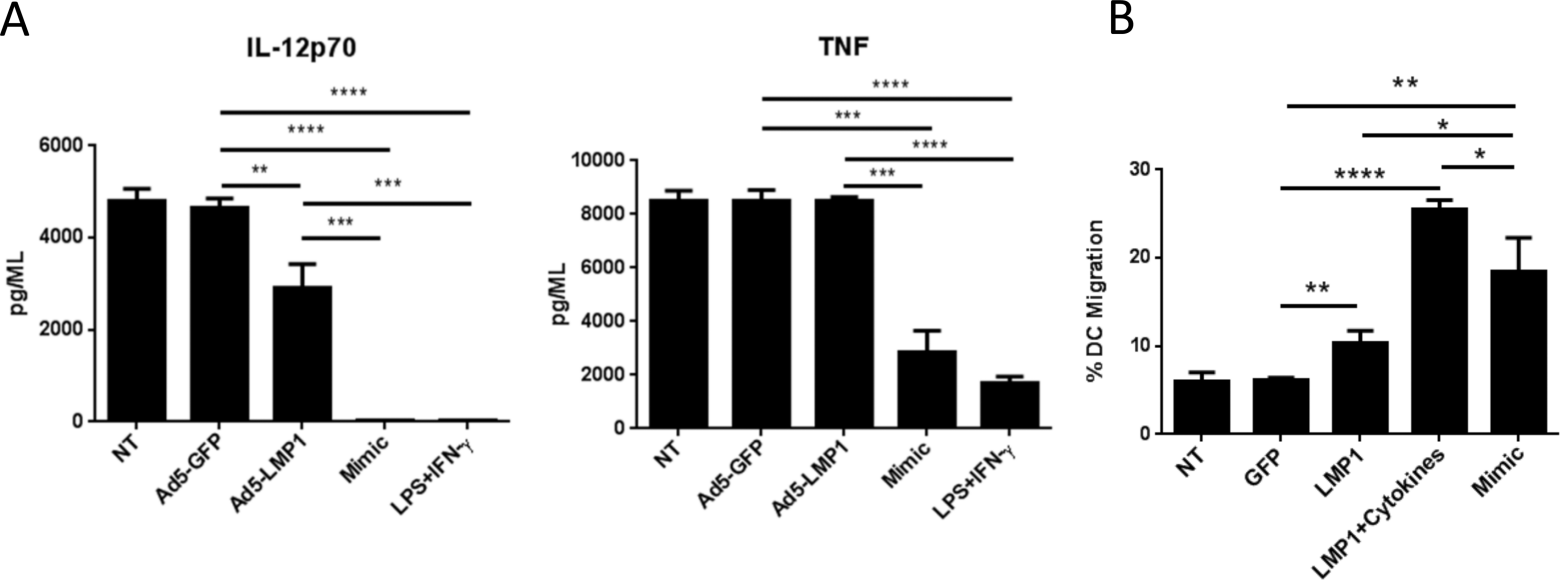
Ad5-LMP1 enhanced migration of human monocyte-derived dendritic cells. Human MDDC were infected with Ad5-LMP1 or Ad5-GFP in the presence of doxycycline. Mimic was added as a positive control. **(A)** After 48-hour maturation, DC were washed and restimulated with LPS and IFN-γ. Supernatants were collected after 24 hours. All samples were analyzed using the BD human inflammation CBA kit. **(B)** MDDC were left untreated or matured with the indicated combinations of AD5 and/or immunostimulatory reagents. After 48 hours culture, DC migration was evaluated using a transwell migration assay in response to CCL19. 1.5x10^5^ DC were allowed 90 minutes to migrate. For the LMP1+Cytokines group, cells were transduced with Ad5-LMP1 and then treated with Mimic cytokines IL-6, IL-1β, and TNF-α in the absence of PGE2. Data represent results from three independent experiments.

A critical step in the induction of an adaptive immune response is the ability of matured DC to secrete IL-12p70 following restimulation by helper T cells in the draining lymph node [31]. Given the low levels of IL-12p70 secretion we observed from LMP1-matured human DC, we decided to evaluate the ability of LMP1-matured DC to secrete IL-12p70 in response to restimulation, and compare this to the capacity of Mimic-matured DC to secrete IL-12p70 following restimulation. First, human DC were stimulated with Ad5-LMP1, Ad5-GFP, Mimic, LPS + IFN-γ, or left untreated. 48 hours later, DC from all groups were restimulated with LPS + IFN-γ. Cytokine levels were measured 24-hours later (Fig 8A). Surprisingly, LMP1-matured DC restimulated with LPS + IFN-γ secreted high levels of IL-12p70 and TNF. IL-12p70 and TNF levels approached that of restimulated immature DC (untreated or Ad5-GFP transduced) (Fig 8A). In contrast, DC stimulated with Mimic or LPS + IFN-γ secreted significantly lower levels of IL-12p70 and TNF compared to LMP1-matured DC following restimulation. These data are consistent with Mimic-induced DC exhaustion that has been previously described following Mimic maturation of DC [17, 18].

### Ad5-LMP1 transduction increased migration of human MDDC in vitro

We next turned to a CCL19 transwell migration assay to assess migration of human DC following Ad5-LMP1 transduction. Previous studies have shown that PGE2 is required for MDDC migration [13, 14]. To evaluate migration by LMP1-matured DC, human MDDC were transduced with Ad5-LMP1 for 48 hours in the presence of doxycycline and then evaluated for migration toward CCL19. Ad5-LMP1 significantly enhanced DC migration compared to Ad5-GFP (p = 0.0078) (Fig 8B). Similarly, Mimic-DC significantly increased migration compared to both GFP-DC and LMP1-DC (p = 0.0056 and p = 0.0279, respectively). Surprisingly, LMP1-matured DC cultured in the presence of the cytokines TNF, IL-1β, and IL-6 (but not PGE2) further enhanced migration to levels significantly higher than that of either Ad5-LMP1 alone (p = 0.0001) or Mimic-matured DC (p = 0.0376) (Fig 8B).

### Ad5-LMP1 transduction enhanced anti-Gag T cell responses in HIV+ patient DC:T cell co-cultures

**Fig 9:**
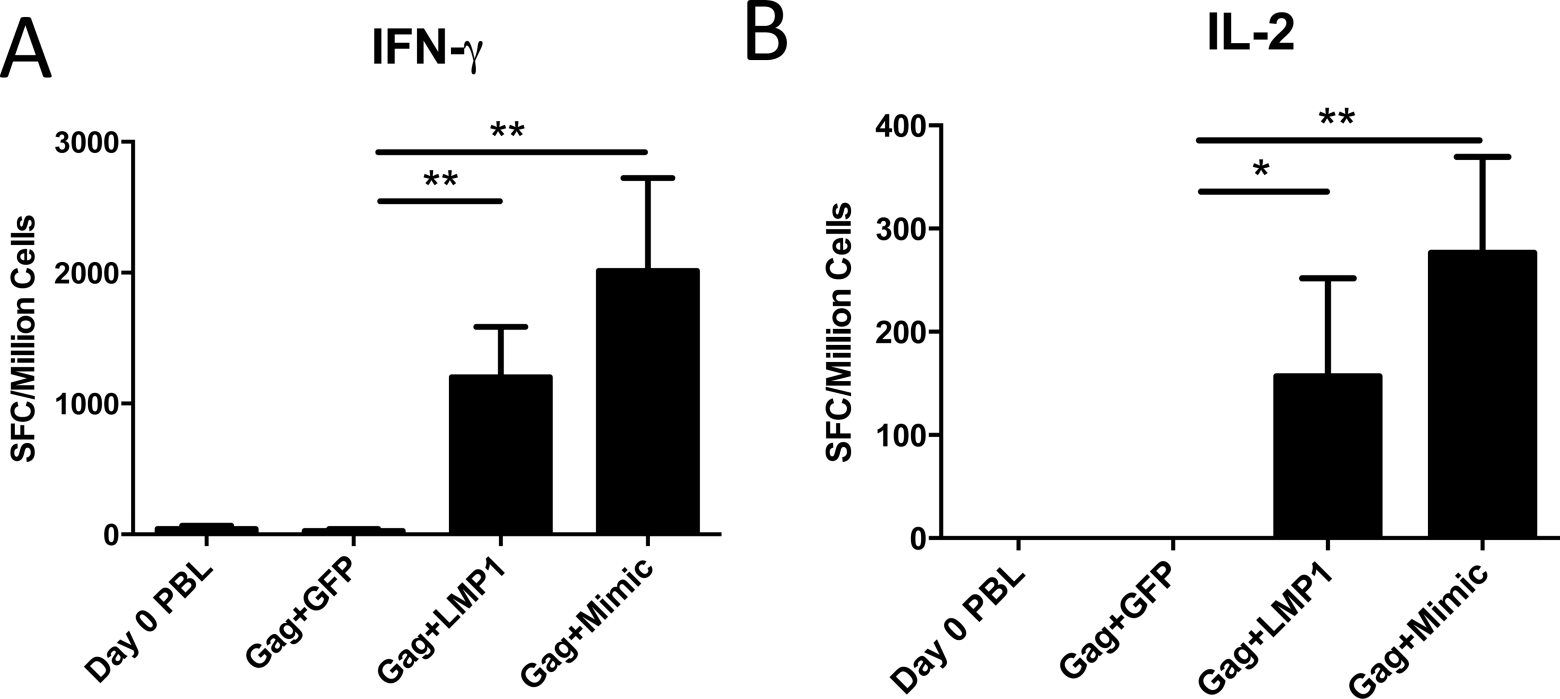
Human HIV+ DC:T cell coculture. MDDC from HIV+ patients on ART were transduced with Ad5-Gag and matured with Ad5-LMP1, Ad5-GFP or Mimic overnight in the presence of doxycycline. DC were then cocultured with autologous patient lymphocytes at a ratio of 10,000DC:100,000 lymphocytes. After 12 days, cells were analyzed by IFN-γ **(A)** or IL-2 **(B)** ELISPOT assay in response to stimulation with a Gag peptide pool. Data represent results from three independent experiments.

Next, we evaluated LMP1-matured DC using an in vitro human model for their ability to stimulate Gag-specific T cell responses, using PBMC from virally-suppressed HIV+ patients on antiretroviral therapy. MDDC generated from patient monocytes were transduced with Ad5-Gag and either Ad5-LMP or Ad5-GFP. As a positive control, DC were matured with Mimic. DC were then cultured with autologous peripheral blood lymphocytes (PBL) for 12 days and T cells evaluated for Gag-specific responses by ELISPOT assay. LMP1-DC significantly increased IFN-γ and IL-2 ELISPOT T cell responses following Gag peptide restimulation compared to untreated lymphocytes (Day 0 PBL) or lymphocytes co-cultured with GFP-transduced DC (Fig 9A and 9B). DC transduced with Ad5-Gag and matured with Mimic induced similar levels of IFN-γ and IL-2 compared to LMP1-DC, however, we observed no significant differences between the two groups, suggesting LMP1-DC are not superior to Mimic-DC in this model.

## Discussion

Immunotherapy with monocyte-derived DC is a promising approach for the treatment of cancer and chronic viral infections such as HIV. Despite this promise, DC immunotherapy clinical trials have only shown modest efficacy to date, possibly due to suboptimal or dysregulated DC maturation and activation [32, 33]. In particular, the chemical PGE2 is known to disrupt the ability of monocyte derived DC to secrete IL-12p70 in response to restimulation [15, 16, 18, 34]. However, PGE2 induces migration of monocyte derived DC to the local draining lymph node, necessitating the use of this chemical in commonly used DC maturation cocktails [13, 14]. We hypothesized that the viral protein LMP1, a protein previously shown by us to activate and mature human DC [23], could induce lymph node migration without the requirement for PGE2. The LMP1 intracellular domain interacts with TRAF1-4, TRAF5, TRADD and RIP and induces NF-κB mediated activation in a manner similar to constitutively active CD40 [21, 35, 36]. While LMP1 has been previously evaluated as an antigen for DC immunotherapy [37], these studies tested a deletion mutant of LMP1 unable to induce immune activation [38]. To our knowledge this report is the first evaluation of full length, immunologically-active LMP1 as a molecular adjuvant for DC immunotherapy. In particular, LMP1 does not require ligand interaction for immune activation, as compared to alternative maturation methods such as transduction with full length CD40. The LMP1 transmembrane domain can also act as a scaffold to induce the constitutive activation of other activation domains, including LMP1 transmembrane domain fusion to the intracellular domain of CD40 or MyD88.

Initial luciferase and SEAP assays (Fig 2) showed that Ad5-LMP1 was a potent inducer of NF-κB and IFN-β expression in vitro. Surprisingly, Ad5-LMP1 transduction was not toxic to DC, in contrast to previous reports with Ad5-LMP1 constructs [38], perhaps reflecting different expression levels between constitutive LMP1 and doxycycline-induced LMP1 expression. Ad5-LMP1 was also effective at maturing and activating both human and mouse DC (Figs 3B and 8B), and induced the secretion of a number of pro-inflammatory cytokines from transduced DC (Figs 3C and 7E). Of note, CD86 showed upregulation following Ad5-LMP1 transduction, despite low levels of CD86 upregulation in previous studies using LMP1 mRNA electroporation [23]. Enhanced CD86 expression may reflect difference in innate immune responses and cell stress when comparing electroporation and viral transduction for gene delivery. IL-12p70 secretion by LMP1-DC was of particular interest. IL-12p70 plays a critical role in the induction of a T cell mediated adaptive immune response [39]. DC maturation in the presence of PGE2 has been previously shown to lead to exhaustion and an inability of DC to secrete IL-12p70 following restimulation [18, 34, 40]. In studies with mouse DC, Ad5-LMP1 transduction was sufficient to induce high levels of IL-12p70 secretion (Fig 3C). In studies with human DC, LMP1 transduction alone induced only moderate levels of IL-12p70 at 48 hours. However, human LMP1-matured DC were able to secrete high levels of IL-12p70 upon restimulation (Fig 8A). In contrast, human Mimic-matured DC failed to secrete IL-12p70, either during Mimic maturation or following restimulation. These findings have important implications for DC immunotherapy. LMP1-matured DC are anticipated to secrete high levels of IL-12p70 following stimulation by CD40L-expressing T cells in the draining lymph node. Restimulation would lead to a synchronized immune response with simultaneous TCR antigen presentation and IL-12p70 secretion by the DC, leading to a robust adaptive immune response. In contrast, Mimic-matured DC are expected to secrete minimal levels of IL-12p70 in response to T cell restimulation, leading to a suboptimal adaptive immune response. One limitation of this study was our failure to include an Ad5-CD40L construct for comparison. A number of groups have shown that CD40L is effective in DC immunotherapy studies [41, 42]. In future experiments we plan to directly compare Ad5-LMP1 to Ad5-CD40L and determine the relative efficacy of these two approaches, as well as any potential synergy with LMP1/CD40L co-administration.

Our data also has important implication for DC migration following DC injection. To date, PGE2 has been a necessary component of DC maturation cocktails, required to promote efficient migration of DC to the lymph node [13, 14]. Alternative DC maturation protocols that do not contain PGE2 can induce migration [27, 43], but typically fail to approach DC migration levels induced by Mimic. In contrast, LMP1 significantly increased in vitro migration of human DC toward CCL19 (Fig 8B), and increased in vivo DC migration in mouse models (Fig 4B). Furthermore, culture of human LMP1-matured DC with cytokines IL-6, IL-1β, and TNF-α increased migration to a level significantly higher than Mimic-matured DC. These findings support the use of LMP1 as a replacement for PGE2, either alone or in combination cytokines IL-6, IL-1β, and TNF-α.

Importantly, LMP1 enhanced adaptive immune responses in both human and mouse models. LMP1-DC stimulated a robust T cell response in a Pmel mouse model (Fig 4C), and slowed tumor growth for both local and metastatic B16-F10 tumors (Fig 5). LMP1-DC failed to fully regress tumors in this model. However, given the aggressive nature of B16-F10 melanoma [44], these findings do support the hypothesis that LMP1-DC enhanced the anti-tumor immune response. Similarly, LMP1-DC generated a 5-log reduction in vaccinia-Gag viral titers in a prophylactic vaccine challenge model following a single injection of a relatively small number of DC (1x10^5^) (Fig 6). Overall, 5 out of 9 animals receiving LMP1-DC showed undetectable virus 5 days post-challenge.

In a human in vitro model, LMP1 significantly increased the generation of antigen-specific T cell responses. Using a HIV in vitro T cell response model, LMP1-matured DC induced a superior Gag-specific T cell response compared to immature DC (Fig 9). However, we observed no statistical difference in IFN-γ and IL-2 ELISPOT responses between LMP1-DC and Mimic-DC, suggesting that both LMP1 and Mimic can induce similar levels of adaptive T cell responses in this human in vitro system. Despite the similar T cell responses induced by LMP1 and Mimic, we anticipate that LMP1-matured DC will be superior to Mimic DC in vivo, given the ability of LMP1-matured DC to induce an IL-12p70 restimulation response. This in vitro HIV immune model did not allow us to evaluate restimulation and will require future studies with in vivo humanized mouse models to test both LMP1 induced DC maturation and the CD4-mediated restimulation of the DC within the lymph node. Future studies should also determine the role of Ad5-induced antigen-specific responses.

An important concern for LMP1 is the oncogenic nature of the protein. LMP1 is involved in the transformation of B cells following EBV infection [45]. Given that ex vivo monocyte-derived dendritic cells are non-dividing cells, the risk of oncogenesis is minimal. Nevertheless, alternative approaches to avoid integration of LMP1 into the genome include an RNA-based approach, such as the use of a non-integrating RNA virus vector (alphavirus or LCMV) for transduction. These viruses remain RNA throughout their replication cycle, avoiding the generation of LMP1 DNA. Another alternative is electroporation or nanoparticle-mediated delivery of mRNA encoding LMP1. RNA-based delivery should minimize the risk to the patient while still allowing LMP1-mediated maturation of DC.

In summary, LMP1 is a promising molecular adjuvant candidate for DC immunotherapy, either alone or in combination with cytokines IL-6, TNF-α, and IL-1β. LMP1 matures and activates DC, induces pro-inflammatory cytokine secretion, and enhances DC migration. LMP1-matured DC secreted high levels of IL-12p70 following restimulation, in marked contrast to Mimic-matured DC. We also observed in vivo efficacy of LMP1-DC in mice, including reduced tumor growth and increased survival, and in vitro efficacy of LMP1-DC in human DC:T cell coculture assays. Overall, this study highlights the potential of LMP1 as a gene-based molecular adjuvant for DC immunotherapy, and as a replacement for DC immunotherapy protocols that require the use of PGE2 for optimal DC migration.
